# Assessment of medical intern’s knowledge, awareness and practice of familial hypercholesterolemia at academic institutes in Jeddah, Saudi Arabia

**DOI:** 10.1186/s12944-020-01266-y

**Published:** 2020-05-21

**Authors:** Sami H. Alzahrani, Abdulhadi Bima, Mohammed R. Algethami, Zuhier Awan

**Affiliations:** 1 Family Medicine Department, Faculty of Medicine, King Abdulaziz University, PO Box 80205, Jeddah, 21589 Saudi Arabia; 2 Clinical Biochemistry Department, Faculty of Medicine, King Abdulaziz University, Jeddah, Saudi Arabia; 3 King Abdulaziz University Hospital, Jeddah, Saudi Arabia

**Keywords:** Cardiovascular diseases, Familial hypercholesterolemia, Medical intern, Knowledge, Awareness, Practices, Gender

## Abstract

**Background:**

Familial Hypercholesterolemia (FH) is a serious under-diagnosed disease characterized by raised low-density lipoprotein cholesterol (LDL-C) and premature coronary artery diseases (CAD). The scarcity of FH reported patients in Saudi Arabia indicates lack of FH awareness among physicians.

**Objective:**

The goal of this research was to assess knowledge, awareness, and practice (KAP) about FH disorder among Saudi medical interns and to identify areas that need educational attention.

**Methods:**

This cross-sectional study involved 170 Saudi medical interns (83 males and 87 females) from academic institutes in Jeddah, Saudi Arabia. The interns were asked to fill an online FH-KAP questionnaire. Total score for each separate domain measured by adding correct answers.

**Results:**

Although, knowledge of FH definition (76.5%) and classical lipid profile (52.4%) were reasonable; knowledge on inheritance (43.5%), prevalence (12.4%) and CAD risks (7.1%) were poor. Knowledge score was significantly higher in female than male (7.5 ± 3 vs. 5.3 ± 2.6, *P* < 0.001). Regarding awareness, 54.1% were familiar with FH disorder, 50.6% with the presence of lipid clinic but only 16.5% were acquainted with guidelines. Furthermore, in the practice domain 82.9% selected statin as first line treatment and 62.9% chose routinely checking the rest of the family, while 15.3% chose ages 13–18 years to screen for hypercholesterolemia in patients with a positive family history of premature CAD.

**Conclusion:**

Substantial defects in FH-KAP among Saudi medical interns were found, emphasizing the importance of professional training. Extensive and constant medical education programs as early as an internship are required to close the gap in CAD prevention.

## Introduction

Since Muller described, back in 1938, xanthomas collections, raised serum cholesterol and myocardial infarction (MI) [1] and later in the 1970s Brown and Goldstein reported the defects in the low-density lipoprotein receptor (LDLR) as the etiology of familial hypercholesterolemia (FH) [2], our understanding of FH has grown tremendously. Familial hypercholesterolemia is a classical monogenic worldwide health disturbance of lipoprotein metabolism manifested by raised serum values of low-density lipoprotein cholesterol (LDL-C) since birth [2, 3]. FH is inherited mainly as an autosomal dominant (AD), but it can also be inherited as an autosomal recessive (AR) pattern. Mutation in one or more genes leads to FH. The well-known AD genes are: low-density lipoprotein receptor (*LDLR*) [2], proprotein convertase subtilisin/ Kexin type 9 (*PCSK9*) [4], and apolipoprotein B-100 (*APOB*) [5]. Alteration in one allele of the above-mentioned genes leads to heterozygous FH (HeFH) while alterations in two alleles cause the severe form of homozygous FH (HoFH). Alteration of LDL receptor adaptor protein 1 (*LDLRAP1*) gene leads to AR type [6]. If FH is left unmanaged, the occurrence of premature coronary artery diseases (CAD) in HoFH patients are definitive, meanwhile, HeFH patients carry a 50% CAD hazard in men and 30% in women by 50 years [2]. The AR-type of FH is clinically similar to HeFH and HoFH with moderate elevations in LDL-C values if the two alleles were altered and the clue is the normal parents’ lipid profile and the dramatic respond to lipid-decreasing modulates [7].

The estimated prevalence of HeFH is 1 in 200 to 500 in many populations [8–10]. with a prevalence of one HoFH in a million individuals [11]. Recent reported HeFH prevalence are 1 in 213, 250, 353 in China, United States and Australia, respectively [9, 10, 12] meanwhile, the problem magnitude is unsettled in the Middle East, including Saudi Arabia [13]. There is an absence of genetic epidemiological research carried to determine FH prevalence in Saudi Arabia [14]. Depending on public census (http://www.stats.gov.sa/en/node), the expected FH prevalence in Saudi Arabia is between 63,485 to 158,712 patients of HeFH, depending on the rate of 1:200–500. The true FH prevalence in Saudi Arabia and Middle East is hindered because of the absence of genetic screening programs and national records [14]. In comparison, three Western countries reported more than 500 gene mutations in FH, while only fifty seven mutations were documented from seventeen Middle Eastern and North African (MENA) countries [15]. Although FH criteria is helpful, some practical limitation exists including lack of family history, clinical manifestations like corneal arcus, xanthelasmas, tendon xanthomas and DNA analysis. Furthermore, engaged clinical settings from primary to tertiary care, preoccupied by available beds and early discharge plans and fail to secure preventive measures to identify and follow-up FH patients [16]. These diagnosis complexities in addition to the high consanguinity rate in the Middle East inflates rates of FH under-reporting and under-management and indicates poor awareness of cardiovascular diseases (CVD) associated with FH [8].

A major debate in clinical practice these days is to elevate FH awareness between physicians so they might increase efforts to identify and treat these patients with intensive LDL-C-lowering drugs. Increasing FH awareness is very important, as most FH patients remain undetected. An additional problem is that current management of FH patients is often suboptimal and, even if treated with available lipid-lowering drugs, patients with FH still have considerably increased CVD mortality versus general population. Increased awareness and a wider urge to treat FH patients appropriately is required to decrease their high risk for premature chronic heart diseases and death [17]. For patients with severe familial hypercholesterolaemia, therapy should be beginning with ezetimibe and statins, and other conventional therapy. If treatment aims didn’t get, new drugs (as PCSK9 suppressors, mipomersen and lomitapide) should be given [18].

The aim of this study was to assess knowledge, awareness, and practice (KAP) about FH disorder among Saudi medical physicians especially during their internship year, to detect if it provides them with good knowledge and practical skills to manage FH cases during their post-qualification.

## Methods

### Study design and setting

This cross-section, questionnaire-dependent work was carried among Saudi medical interns at two academic institutions: King Abdulaziz University and University of Jeddah, Saudi Arabia during the month of April 2019. This study was accepted by the biomedical ethical committee at the two universities and all doctors were notified about the research objectives. Written informed consent was optioned from participants before entering the research. Permission was taken from Sjouke et al. to use the questionnaire [19]. The questionnaire was validated and distributed as an electronic form. FH-KAP questionnaire validity was tested using PCPPG- Qual test [20] and reliability coefficient was tested using KR-20 reliability coefficient [21]. The questionnaire is composed of two parts: the first one inquired about demographic characteristics and consists of 3 questions (age, gender, and GPA score) and the second part inquired about knowledge, awareness, and practice in FH (FH-KAP) disorder and consists of 20 items with a total of 38 marks.

### Study instrument

The FH-KAP questionnaire utilized in this research was initially made by Bell et al. [22] It contained 20 items cover wide items of FH-KAP, 10 knowledge (17 marks), 5 awareness (13 marks) and 5 practices items (8 marks). These items found in many designs as single best answer question, 7-point Likert scale, dichotomous answers of ‘Yes / No / Don’t know’, multiple answer questions, and make out answer statement. The participants asked to choose the right answer. They could select more than one answer in many questions. The FH questions evaluate participant’s knowledge of FH as clinical properties, diagnostic lipid profile, mode of inheritance, prevalence, awareness of genetic cause for the diagnosis and relation of FH with CAD. Some sections assess intern’s awareness of new guidelines, therapeutic alternatives and patients’ treatment goals. They were also assessed for investigations that helped in screening for possible FH and reflect best medical practice in early detection.

### Questionnaire score

The FH-KAP score was made according to a published study [23]. For determined FH familiarity, Likert-scale, a seven points scale in which one denotes “not at all familiar” and seven denotes “extremely familiar”, results between 1 and 4 meant ‘unfamiliar’ and encoded ‘0’, meanwhile results of 5–7 meant ‘familiar’ and encoded as ‘1’. Incorrect answer encoded as ‘0’, while right answer coded as ‘1’. The questionnaire score was made by adding the right responses of all questions in each domain. Total score mark for every individual ranged from 0 to 19 for knowledge, 0–15 for awareness, and 0–9 for practices. Each domain total scores range were un-similar with items numbers as some statements had more than one right answer. The mean of total KAP score of ≥50% for each domain was acceptable [24].

### Sample size calculation

The numbers of medical interns at the two academic institutions in the year 2019 were 230 doctors. The sample size was made by RAOSOFT calculator (http://www.raosoft.com/samplesize.html), assuming 50% probability, 95% confidence level, and 5% sampling error. The minimum measured sample was 145. Presuming non-response rate of 15%, the sample size was elevated to 170. The sample was proportionally stratified based on gender.

### Subject recruitment and data collection

One hundred and seventy out of 230 (73.9%) Saudi medical interns at the two academic institutions completed the online survey and were recruited via simple random sampling by digits from doctors’ names lists. Participants were screened for competence based on exclusion and inclusion criteria. Medical students from, general practicing (GP) and physicians were excluded. Eligible medical interns were asked to fill an online questionnaire in about fifteen minutes without returning to notes or online recourses. The completed sheet was returned to researchers for analysis.

### Statistical analysis

Data analysis was done by SPSS software version 20 (SPSS Inc., Chicago, IL, USA). Categorical variables were reported as frequency and percentage (%) and parametric parameters as mean +/− standard deviation (SD) and minimum and maximum values. The Chi-Square test was used to evaluate the dissimilarity of correct answers between males and females. The normality of data was assessed by Shapiro- Wilk test, and accordingly, the total score for knowledge, awareness, and practice domains showed abnormal distribution, thus Mann Whitney test was utilized to differentiate mean scores between males and females. While the age was normally distributed so unpaired student “t” test was applied for comparison. A *P*-value of < 0.05 indicated significant changes.

## Results

### Demographic and practice details

The sheets were given to 170 Saudi medical intern doctors, 83 (48.8%) were males and 87 (51.2%) were females. Their age ranges from 23 to 32 years old. The age of males was significantly higher than females (*P* = 0.0001). The GPA score of all participants is as the following: mostly 3.50–4.49 (66.5%), then > 4.50 (23.5%) and lastly 2.50–3.49 (10.0%). There were insignificant differences in the different grades of GPA scores between males and females (Table 1).

**Table 1:** Demographic characteristics of Saudi Medical interns (*n* = 170)

Characteristics	Total (n = 170)	Male (*n* = 83, 48.80%)	Female (*n* = 87, 51.20%)	*P*- Value
Age (years)	24.36 ± 0.94 (23.00–32.00)	24.70 ± 1.07 (23.00–32.00)	24.03 ± 0.66 (23.00–27.00)	0.0001
GPA				
> 4.50	40 (23.50%)	17 (20.50%)	23 (26.40%)	0.343
3.50–4.49	113 (66.50%)	56 (67.50%)	57 (65.50%)	0.925
2.50–3.49	17 (10.00%)	10 (12.00%)	7 (8.00%)	0.467

Data are expressed as mean+/− standard deviation (minimum – maximum) or number (%) as appropriate. Comparison between male and female was made using Chi-Square test for non – parametric parameters and unpaired student “t” test for parametric parameters

### Knowledge regarding FH

The numbers of correct answers to knowledge items are stratified to male and female and are shown in Table 2. 100.0% of participants correctly answered that none of the features mentioned exclude FH diagnosis, 76.5% correctly defined FH, 52.4% correctly defined FH lipid profile, 45.9% correctly indicated that genetic evaluation is not needed for the right FH diagnosis, 43.5% correctly identified FH mode of inheritance, 12.4% correctly identified FH prevalence, 7.1% correctly identified CAD risk in FH and 4.7% correctly identify target LDL-C value in FH. Regarding family history to be taken in FH, 62.4 select onset age of premature CHD, 52.4% select family history of hypercholesterolemia, 44.7% select family history of tendon xanthomata, 42.9% select family history of childhood unexplained death, 28.8% select the presence of consanguinity, 11.2% select completion of three-generation pedigree chart. Regarding correctly answered that both together statin and ezetimibe is highly advocated for adult HeFH, 37.6% selected Ezetimibe co-administered with statin therapy is recommended as an option for adult heterozygous FH, 10.0% select lipid lowering drug therapy is considered by the age of 10 years, 7.6% select the progress of cascade screening in FH patients should be recorded. The percentage of the female was significantly higher than male in many of the corrected answers Table 1. The mean total score of knowledge was 6.4 and was significantly higher in females than males (7.5 ± 3.0 versus 5.3 ± 2.6, *P* = 0.0001). Only 20.6% had acceptable knowledge of FH while 79.4% of the participants had poor knowledge. The number of females who possess acceptable knowledge was significantly higher than males (P = 0.0001), meanwhile, insignificant difference between males and females with poor knowledge (*P* = 0.102).

**Table 2:** Comparison of overall correct responses in knowledge regarding FH between male and female Saudi medical interns. (10 questions, total score 17)

No	Areas of KAP regarding FH being tested	Total (*n* = 170)	Male (*n* = 83)	Female (*n* = 87)	*P* value
Knowledge items					
3	Correctly defined FH	130 (76.50%)	55 (66.30%)	75 (86.20%)	0.002
4	Correctly defined lipid profile of FH	89 (52.40%)	27 (32.50%)	62 (71.30%)	0.0001
6	Correctly identified FH prevalence	21 (12.40%)	12 (14.50%)	9 (10.30%)	0.281
7	Correctly identified FH inheritance	74 (43.50%)	23 (27.70%)	51 (58.60%)	0.0001
8	Correctly identified CAD risk in FH	12 (7.10%)	5 (6.00%)	7 (8.00%)	0.416
11	Correctly identified that genetic test is not required for accurate FH diagnosis	78 (45.90%)	33 (39.80%)	45 (51.70%)	0.079
22	Correctly identified target LDL-c level in FH	8 (4.70%)	5 (6.00%)	3 (3.40%)	0.334
23	Correctly selected family history of premature CAD to be taken in FH				
	*Consanguinity*	49 (28.80%)	27 (32.50%)	22 (25.30%)	0.191
	*Family history of premature CHD (age of onset)*	106 (62.40%)	38 (45.80%)	68 (78.20%)	0.0001
	*Family history of hypercholesterolaemia (TC and/ or LDL-c)*	89 (52.40%)	37 (44.60%)	52 (59.80%)	0.034
	*Family history of tendon xanthomata*	76 (44.70%)	26 (31.30%)	50 (57.50%)	0.0001
	*Family history of childhood unexplained death*	73 (42.90%)	17 (20.50%)	56 (64.40%)	0.0001
	*Three-generation pedigree chart*	19 (11.20%)	11 (13.30%)	8 (9.20%)	0.276
24	Correctly responded that none of the features given would lead to exclusion of FH diagnosis	170 (100.00%)	83 (100.00%)	87 (100.00%)	–
25	Correctly identified that combined statin with ezetimibe is recommended for adult HeFH				
	*Ezetimibe co-administered with statin therapy is recommended as an option for adult heterozygous FH*	64 (37.60%)	26 (31.30%)	38 (43.70%)	0.066
	*Lipid lowering drug therapy is considered by the age of 10 years*	17 (10.00%)	9 (10.80%)	8 (9.20%)	0.459
	*Progress of cascade screening in FH patients should be recorded*	13 (7.60%)	5 (6.00%)	8 (9.20%)	0.314
	Overall knowledge of FH (Total Score 17)	6.40 ± 3.01 (1.00–12.00)	5.29 ± 2.63 (1.00–12.00)	7.46 ± 2.98 (1.00–12.00)	0.0001
	Acceptable knowledge (≥ 9.5)	35 (20.60%)	6 (7.20%)	29 (33.30%)	0.0001
	Poor knowledge (< 9.5)	135 (79.40%)	77 (92.80%)	58 (66.70%)	0.102

FH: familial hypercholesterolemia. Data are expressed as mean+/− standard deviation (minimum – maximum) or number (%) as appropriate. Comparison between male and female was made using Chi-Square test for non – parametric parameters and Mann Whitney test for parametric parameters. A mean knowledge score was computed by summing correct answers to all 11 knowledge questions. The knowlege regarding FH was considered acceptable if the total score was ≥50%

### Awareness regarding FH

The numbers of correct answers to awareness items are stratified to male and female and are shown in Table 3. 54.1% are familiar with FH, 50.6% are aware of lipid clinics. Regarding awareness of guidelines; 16.5, 15.3, 9.4, 6.5, 5.9% were acquainted with NICE recommendation, European Atherosclerosis Society Position Paper, International FH Foundation, National Lipid Association and The Japanese recommendations for the Management of FH, respectively.

**Table 3:** Comparison of overall correct responses in awareness regarding FH between male and female Saudi medical interns. (5 questions, total score 11)

No	Areas of KAP regarding FH being tested	Total (*n* = 170)	Male (*n* = 83)	Female (*n* = 87)	*P* value
Awareness items					
1	Familiar with FH	92 (54.10%)	41 (49.40%)	51 (58.60%)	0.146
2	Aware of NICE Guideline on FH	28 (16.50%)	13 (15.70%)	15 (17.20%)	0.472
15	Aware of lipid specialist clinic	86 (50.60%)	28 (33.70%)	58 (66.70%)	0.0001
19	Aware of other international FH guidelines				
	*Integrated Guidance on the Care of Familial Hypercholesterolaemia by the International FH Foundation (2014)*	16 (9.40%)	12 (14.50%)	4 (4.60%)	0.025
	*Homozygous Familial Hypercholesterolaemia: New Insight and Guidance for Clinicians to improve Detection and Clinical Management. A Position Paper from the Consensus Panel on Familial Hypercholesterolaemia of the European Atherosclerosis Society (EAS) (2014)*	26 (15.30%)	21 (25.30%)	5 (5.70%)	0.0001
	*The Japanese Guidelines for the Management of Familial Hypercholesterolemia (Harada-Shiba, 2012)*	10 (5.90%)	9 (10.80%)	1 (1.10%)	0.007
	*Clinical Guidance by the National Lipid Association (NLA) Expert Panel on Familial Hypercholesterolaemia (2011)*	11 (6.50%)	10 (12.00%)	1 (1.10%)	0.004
	*Others*	0 (0.00%)	0 (0.00%)	0 (0.00%)	1.000
20	Aware of FH diagnostic criteria				
	*Dutch Lipid Clinic Network Criteria*	*22 (12.90%)*	*17 (20.50%)*	*5 (5.70%)*	0.004
	*Simon Broome Register Criteria*	*12 (7.10%)*	*11 (13.30%)*	*1 (1.10%)*	0.002
	*US MED-PED Criteria*	*11 (6.50%)*	*8 (9.80%)*	*3 (3.40%)*	0.091
	*Japanese Criteria*	*8 (4.70%)*	*7 (8.40%)*	*1 (1.10%)*	0.027
	*Others*	0 (0.00%)	0 (0.00%)	0 (0.00%)	1.000
	Overall awareness of FH (total score 13)	1.89 ± 1.63 (0.00–6.00)	2.13 ± 1.52 (0.00–6.00)	1.67 ± 1.16 (0.00–6.00)	0.068
	Poor awareness (< 7.5)	170 (100.00%)	83 (100.00%)	87 (100.00%)	1.000

FH: familial hypercholesterolemia. Data are expressed as mean+/− standard deviation (minimum – maximum) or number (%) as appropriate. Comparison between male and female was made using Chi-Square test for non – parametric parameters and Mann Whitney test for parametric parameters. A mean awareness score was computed by summing correct answers to all 5 awareness questions. The awareness regarding FH was considered acceptable if the total score was ≥50%

Regarding diagnostic criteria, 12.9, 7.1, 6.5, 4.7% were familiar with Dutch Lipid Clinic Network Criteria, Simon Broome Register Criteria, US MED-PED Criteria, and The Japanese Criteria, respectively.

While, females were more aware of lipid clinics (*P* = 0.0001), males were significantly higher than females in adopting international FH guidelines and are aware of FH diagnostic criteria. Unfortunately, all participants had poor overall awareness of FH (Table 3).

### Practice regarding FH

The numbers of correct answers to Practice items are stratified to male and female and are shown in Table 4. 82.9% selected statin to treat hypercholesterolemia, 62.9% routinely screened family members in FH diagnosed patients, 38.8% selected statin and ezetimibe combination for severe hypercholesterolemia treatment, 15.3% chose the age range in-between 13 to 18 years old to investigate for hypercholesterolemia if they had a positive family history of premature CAD.

**Table 4:** Comparison of overall correct responses in practice regarding FH between male and female Saudi medical interns. (5 questions, total score 8)

No	Areas of KAP regarding FH being tested	Total (*n* = 170)	Male (*n* = 83)	Female (*n* = 87)	*P* value
Practice items					
10	Screened premature CAD patients for FH including screening the family				
	*Look for arcus cornealis*	*25 (14.70%)*	*16 (19.30%)*	*9 (10.30%)*	0.076
	*Look for tendon xanthomata*	*42 (24.70%*	*25 (30.10%)*	*17 (19.50%)*	0.078
	*Take a detailed family history of coronary heart disease*	*45 (26.50%)*	*25 (30.10%)*	*20 (23.00%)*	0.190
	*Screen close relatives for hypercholesterolaemia*	*35 (20.60%)*	*20 (24.10%)*	*15 (17.20%)*	0.180
12	Routinely screened family in FH patient	107 (62.90%)	40 (48.20%)	67 (77.00%)	0.0001
14	Selected age 13–18 years to test young individuals for hypercholesterolaemia if they have family history of premature CAD	26 (15.30%)	18 (21.70%)	8 (9.20%)	0.020
17	Selected statin to treat hypercholesterolaemia	141 (82.90%)	62 (74.70%)	79 (90.80%)	0.005
18	Selected combination of statin and ezetimibe for treatment of severe hypercholesterolaemia	66 (38.80%)	31 (37.30%)	35 (40.20%)	0.410
	Overall practices of FH (Total Score 8)	2.86 ± 1.71 (0.00–8.00)	2.86 ± 1.85 (0.00–8.00)	2.87 ± 1.85 (0.00–8.00)	0.846
	Acceptable practice (≥ 4.5)	25 (14.70%)	13 (15.70%)	12 (13.80%)	0.841
	Poor practice (< 4.5)	145 (85.30%)	70 (84.30%)	85 (86.20%)	0.678

FH: familial hypercholesterolemia. Data are expressed as mean+/− standard deviation (minimum – maximum) or number (%) as appropriate. Comparison between male and female was made using Chi-Square test for non – parametric parameters and Mann Whitney test for parametric parameters. A mean practice score was computed by summing correct answers to all 6 practice questions. The practice regarding FH was considered acceptable if the total score was ≥50%

The corrected responses of all participants in regard to screening the families of FH patients, were 26.5, 24.7, 20.6, 14.7% by taking a detailed family history of CAD, looking for tendon xanthomata, screening close relatives for hypercholesterolemia, and looking for arcus cornealis, respectively.

The total score of the practice was 2.86 out of 8 and there was an insignificant difference between male and female (2.86 ± 1.9 versus 2.9 ± 1.9, *P* = 0.846). In all participants, 14.7% had acceptable practice, while 85.3% had a poor practice of FH (Table 4).

### Opinion about health care providers

Regarding opinion about health care providers that would be most useful in early FH detection and screening first-degree relative revealed that most of our intern doctors select GPs (60%) followed by lipid specialists, endocrinologists, cardiologists, pediatricians, Obstetricians/ Gynecologists and lastly nurses with experience in cardiac risk prevention (11.8,10.6,10.6,4.7,1.8, 0.6%, respectively) (Table 5)

**Table 5:** Opinion about health care providers would be most effective at early detection of FH and screening first-degree relatives

Areas of KAP regarding FH being tested	Total (*n* = 170)	Male (*n* = 83)	Female (*n* = 87)	*P* -value
General practitioners	102 (60.00%)	32 (38.60%)	70 (60.00%)	0.001
Lipid specialists	20 (11.80%)	15 (18.10%)	5 (5.70%)	0.025
Endocrinologists	18 (10.60%)	13 (15.70%)	5 (5.70%)	0.059
Cardiologists	18 (10.60%)	11 (13.30%)	7 (8.00%)	0.346
Pediatricians	8 (4.70%)	8 (9.60%)	–	–
Obstetricians/ Gynecologists	3 (1.80%)	3 (3.60%)	–	–
Nurses with experience in cardiac risk prevention	1 (0.60%)	1 (1.20%)	–	–

Data was expressed as number (%). Comparison between male and female was made using Chi-Square test

.

## Discussion

Cardiovascular diseases are the main cause of mortality and disease burden all over the world [25]. Medical intern doctors are in a good position to detect undiagnosed FH cases due to their first encounter with the patients without the intricacy of a busy routine practice. The high consanguinity rates in Arabian Gulf (Saudi Arabia, United Arab Emirates, Oman, Kuwait, Qatar) (up to 50%) [26] and absence of national registries and screening programs [15] suggests that FH prevalence may be more than what is reported. Therefore, a cross-national Gulf FH-Registry began in February 2017. This initiative supported the opening of lipid clinics and lipoprotein apheresis centers. Sadly, only 13% of cases with acute coronary syndrome get LDL-C less than 70 mg/dL (Gulf-COAST registry) indicating a gap in medical knowledge and practice [27].

Unfortunately, this survey revealed that only 20.6% of medical interns had acceptable FH knowledge. Acceptable knowledge was reported in FH description (76.5%) and lipid profile that diagnosed FH (52.4%). This is in accordance with previous researches among primary care physicians (PCP) who rightly define FH and know FH specific lipid levels in Malaysian (61.6 and 77.7%) [28], Western Australia (80 and 68%) [22], United Kingdom (89 and 74%) [29] and Asia Pacific areas (86 and 65%) [24]. On the contrary, a decreased number of PCP in Southern India accurately define FH and know specific lipid levels (71 and 35%) [30].

The medical interns in this study had poor knowledge regarding global FH prevalence (12.4%), inheritance (43.5%), CAD risk (7.1%), genetic test (45.9%), target LDL-C reduction following maximum tolerance dose of high-intensity statin for diagnosed FH patients (4.7%), important knowledge obtained from family history in FH patients as consanguinity (28.8%), tendon xanthomata (44.7%), unexplained death (42.9%), three-generation pedigree chart (11.2%) and also poor knowledge regarding FH patients management options (37.6%), giving lipid-lowering drug therapy by age of 10 years (10.0%), recommendation of cascade screening progression in FH patients (7.6%). Many studies reported physicians’ FH-KAP to be suboptimal [22, 29–31]. In Malaysia, among 193 PCP, only 23.8, 66.8, and 40.4% gave correct answers to global prevalence, FH clinical features, and transmission rate to first degree relatives, respectively [32]. Identification of the pattern of FH inheritance among physicians was 47% in Asia [31], 50.50% in UK [29] and 33% in Riyadh, Saudi Arabia [23]. The most concerning is PCP capability to know high CAD hazard in untreated FH was only 8.3% respond rightly in Malaysian [28], 9% in Asia Pacific region [24], 14% in UK [22], 14% in Southern India [30] and 29% in Western Australia [29]. This may be explained partially by the under-detection of FH patients.

International roles marked FH patients as possess elevated cardiovascular hazards; so, optimal LDL-C goal must be < 2.5 mmol/L or < 1.8 mmol/L with proven atherosclerotic CVD or at least a 50% decrement in LDL-C values [8, 33]. In this study, only 4.7% of Saudi medical interns were oriented with optimal LDL-C values of FH patients. In this respect, over half of physicians in Batais et al. [23] study among 294 physicians in four big hospitals in Riyadh, Saudi Arabia was not known LDL-C values of FH cases. In Centralized Pan-Middle East Survey (CEPHEUS) that was made in 6 Persian Gulf regions, 52% of cases get their LDL-C targets [34]. There is poor adherence of the physicians to general dyslipidemia recommendations [35].

Early therapy must be initiated in FH cases, as they possess about 20-fold more hazards of getting premature CAD [36]. In this study, only 7.1% know that CVD hazard in unmanaged FH cases as 20 times more compared to general people. In Batais et al. [23] survey among physicians in Riyadh, Saudi Arabia, > 90% of physicians were fit to detect CVD hazard in unmanaged FH individuals as 20 times more than general peoples, but were unable to know the age threshold for premature CAD in male and female.

Good family history is essential to CVD hazard check among FH. Pang et al. [24] reported that 90% of PCPs took complete family history in premature CAD cases. European roles propose checking children in FH kindred from 5 years old age [37] and NICE instructions advocate checking children between 2 and 10 years, PCPs in Asia-Pacific area suggested screening between 13 and 18 years old more convenient [24].

In this study, the percentage of the female were higher than male in many of the answers related to knowledge questions in defining FH, lipid profile, FH inheritance, family history of premature CHD (age of onset), family history of hypercholesterolemia (TC and/ or LDL-C), positive family history of tendon xanthomata, and family history of childhood unexplained death. The mean of the total score of knowledge was significantly higher in females than males. This finding can partially be explained by elevated GPA score of females than males in the undergraduate years as well as the teaching setting is been conducted in independent campuses and independent staff for each gender, so the knowledge they acquired may vary. Batais et al. [24] found that FH knowledge scores more between physicians with longer duration in practice and those managed FH cases. These results suggested that future physicians’ training may improve their ability to effectively treated FH.

Unfortunately, all Saudi medical interns (100.0%) had poor awareness about FH. Acceptable awareness was only reported about familiarity with FH (54.1%) and awareness of lipid specialist clinic (50.6%). Meanwhile, the awareness gap was reported regarding international FH guidelines and FH diagnostic criteria. In this study, the correct response was higher in female than male in awareness of lipid specialist clinic, while, awareness was lower in female than male of other international FH roles and FH diagnostic criteria. There are several causes of this poor awareness. The clinical symptoms and signs of FH are not common; a family history of raised LDL-C is difficult to detect. The diagnostic criteria for FH rely on Dutch Lipid Clinics Network or Simon Broome criteria [38], might had been taught in medical school but were quickly forgotten [39]. The poor awareness of guidelines and lipid specialists can be due to the absence of country-specific guidelines [40] on FH and the poor training of physicians in the field of clinical Lipidology.

Many criteria are present to screen FH cases, as Make Early Diagnosis to Prevent Early Death [41], Simon Broome [42], DLCN criteria [43], US MED-PED criteria and Japanese criteria [44]. While, these methods could help to discover FH individuals, the DLCN guidelines commonly used due to their higher sensitivity [33, 45]. Genetic testing could be used in FH diagnosis and to determine the type of FH (heterozygous or homozygous) [46]. In Malaysia, decrease awareness (< 20%) between PCP of different clinical algorithm methods was found with 25.9% know of cascade checking for FH cases [23]. In another study in Malaysia, defects were present in awareness of FH clinical guidelines between PCP. Less than half of the participants were aware of NICE roles (39%) and their awareness of other international FH roles was less (19.1%) and 72.8% of Malaysian PCP were unaware of FH diagnostic stigmata [28]. In the UK, 43.5% of physicians were aware of Simon Broome diagnostic stigmata and 65.9% were aware that family cascade checking is needed by NICE instructions [29], 61% of PCP was aware of NICE instructions on FH [24]. This is very important as cascade checking of first-degree relatives of FH individuals is proven to be the most cost-reliable way to discover undetectable FH patients [47].

The results of this study revealed that acceptable practice was reported in only 14.7% of Saudi medical interns. The acceptable practice was found in a routine check of FH patient’s close relatives with a lipid status (62.9%), selection of statin to treat hypercholesterolemia (82.9%). While, the poor practice was reported in family checking of FH in patients with documented premature CAD, the age for FH checking in young individuals for hypercholesterolemia in a family with premature CAD (15.3%), selected both statin and ezetimibe together for therapy of severe hypercholesterolemia (38.8%). In this study, the correct response of the female was higher than male in routinely check family in FH patient, selection of statin to treat hypercholesterolemia. While the correct answers of the male were higher than female in selection age 13–18 years to check young individuals for hypercholesterolemia with a positive family history of premature CAD. The treatment aim in FH children, the European consensus on FH recommends in children LDL-C < 135 mg/dL (≈3.5 mmol/L) as aim in both heterozygous and homozygous FH, irrespective of age [48]. The presence of very high LDL-C or more cardiovascular risk factors may decrease this target level or the starting age of therapy with statin. In FH children such a target value is hard to reach with available cholesterol-lowering drugs. So, much is expected from the agents against PCSK9, antisense oligonucleotides against apolipoprotein B, microsomal triglyceride transfer protein suppressors, and so on to reduce LDL-C below levels obtained with statin alone [49]. The long-term safety and efficacy results of trials with these medications in children are still under trial [50]. The International FH Foundation offers guidance on FH treatment [33]. These systemic guidelines were lodged as an excellent method for the creation of MENA FH registry [15]. In countries with a history of dedicated checking programs, as Netherlands and Norway, the outcomes in terms of newly diagnosed FH index cases and cascade tested relatives were higher than countries lacking any formal checking program (usually< 1%) [8, 51].

Dissimilarity in the selection of healthcare professional recognized as a better place for treating FH and family checking between different areas might indicate, unlike healthcare programs. In this survey, most medical intern doctors choose GP (60%) as the most effective health care providers to consult rather than lipid specialists (11.8%), endocrinologists (10.6%), cardiologists (10.6%), pediatricians (4.7%), Obstetricians/ Gynecologists (1.8%) and lastly nurses with experience in cardiac risk prevention (0.6%). In Malaysia, the majority of PCP (85.2%) choose themselves as most beneficial healthcare donors in rapid FH diagnosis and family check [28]. In China, most of PCP (83%) think that lipid specialists were best to manage FH [24]. Others view that cardiologists are well-positioned to identify FH cases presenting with coronary artery diseases [37]. Endocrinologists were thought to be in a good position to detect FH in secondary prevention settings [24]. However, due to the large number of GPs in Saudi Arabia it makes perfect sense that they should be consulted first giving that they acquire the proper training well and the complex cases should be referred to lipidologist in other to specialty. Little suggests an important role for nurses [24] which can be explained by the lack of nurses practitioner in this field. This differs from Netherlands [52] results were screening programs have been applied by allied health members and/or nursing. Screening may also be made in a non-medical place as schools and workplaces; this option was not checked in this work. Further detection of health services and systems are needed to optimize country-special clinical service models and care integration [33]. Primary care plays an important role but the absence of infrastructure and supports offered by hospital lipid clinics. Such support will be important if a sustainable primary care-based model of care is established [53].

### Strength and limitation of this study

The strength of this research was the utilization of a validated FH-KAP questionnaire. This study added novel information in the areas of medical education, and included a significant difference between male and female FH-KAP scores that might be a reflection of the medical education system style. This work is limited due to the nature of survey based research and the results cannot proof or disproof causality, where results showed relations and not certainly causal association. Also, medical interns were from two academic institutes in Jeddah, and therefore the results may not be generalized. However, no similar research was made on medical interns and so these results are generally interesting and of great value to improve the medical education system. Another restriction of the present work was that other CVD hazard factors were not surveyed like; hypertension, type 2 diabetes mellitus, obesity and smoking, especially with the rising incidence of risk factors in the Middle East [54].

## Conclusion and recommendations

Our survey suggested that there is a deficit in knowledge, awareness, and practice about FH among Saudi medical interns at King Abdulaziz University and University of Jeddah graduates. Overall, Saudi medical interns had good knowledge in the definition of FH and knowing FH lipid status, familial pattern, aware of lipid specialist clinic and treatment with a statin. However, substantial gaps were found in many items of FH care as knowledge about prevalence, mode of inheritance and CAD hazard factors, genetic test, LDL-C level, premature CAD family history, awareness of clinical roles and characteristics of diagnosis, as well as practice on CAD hazard stratification, FH cases screened family. Female Saudi medical interns had better knowledge regarding FH than males while the male had better awareness aspects than female. Nevertheless, both genders had a low score level of FH-KAP. Our findings support the need for the development of Saudi clinical guidelines for FH management. There is a need for enhancing FH and related lipid topics in medical curricula and restructuring continuing medical education activities at teaching hospitals aimed at medical intern doctors. These strategies can improve the care and detection of FH and decrease the burden of premature CAD.

## Data Availability

All original data is available in the Department of Family Medicine, King Abdulaziz University, Jeddah, Saudi Arabia.

## Abbreviations

FH: Familial Hypercholesterolemia
LDL-C: Low-density lipoprotein cholesterol
CAD: Premature coronary artery diseases
MI: Myocardial infarction
LDLR: Low-density lipoprotein receptor
AD: Autosomal dominant
AR: Autosomal recessive
PCSK9: Proprotein convertase subtilisin/ Kexin type 9
APOB: Apolipoprotein B-100
HeFH: Heterozygous FH
HoFH: Homozygous FH
LDLRAP1: LDL receptor adaptor protein 1
MENA: Middle Eastern and North African
FH-KAP: Knowledge, awareness, and practice in FH
